# Supplementary material for: A fault-intrusion-tolerant system and deadline-aware algorithm for scheduling scientific workflow in the cloud
Source: PeerJ Comput Sci. 2021 Nov 2;7:e747. doi: 10.7717/peerj-cs.747 (PMC8576554; doi:10.7717/peerj-cs.747)
Supplement: Supplemental Information 6 [file peerj-cs-07-747-s006.zip › Myproject1.1/docs/org/workflowsim/clustering/package-summary.html]

org.workflowsim.clustering


JavaScript is disabled on your browser.


- Overview
- Package
- Class
- Use
- Tree
- Deprecated
- Index
- Help

- Prev Package
- Next Package

- Frames
- No Frames

- All Classes

# Package org.workflowsim.clustering

- Interface Summary

  | Interface | Description |
  | --- | --- |
  |  |  |
  | --- | --- |
  | ClusteringInterface | The ClusteringInterface for all clustering methods |
- Class Summary

  | Class | Description |
  | --- | --- |
  |  |  |
  | --- | --- |
  | AbstractArrayList | This data structure AbstractArrayList is used in clustering alone It is better than ArrayList since it associates with depth information |
  | BasicClustering | The default clustering does no clustering at all, just map a task to a job |
  | BlockClustering | BlockClustering groups tasks in both horizontal and vertical direction |
  | HorizontalClustering | HorizontalClustering merges task at the same horizontal level |
  | TaskSet | TaskSet is a group of tasks used only in clustering |
  | VerticalClustering | VerticalClustering merges tasks at the same pipeline |

- Overview
- Package
- Class
- Use
- Tree
- Deprecated
- Index
- Help

- Prev Package
- Next Package

- Frames
- No Frames

- All Classes
